# Percutaneous closure of atrial septal defects leads to normalisation of atrial and ventricular volumes

**DOI:** 10.1186/1532-429X-10-55

**Published:** 2008-12-01

**Authors:** Karen SL Teo, Benjamin K Dundon, Payman Molaee, Kerry F Williams, Angelo Carbone, Michael A Brown, Matthew I Worthley, Patrick J Disney, Prashanthan Sanders, Stephen G Worthley

**Affiliations:** 1 Cardiovascular Research Centre, Royal Adelaide Hospital and The University of Adelaide, Adelaide, South Australia, Australia; 2 The University of Adelaide, Adelaide, South Australia, Australia

## Abstract

**Background:**

Percutaneous closure of atrial septal defects (ASDs) should potentially reduce right heart volumes by removing left-to-right shunting. Due to ventricular interdependence, this may be associated with impaired left ventricular filling and potentially function. Furthermore, atrial changes post-ASD closure have been poorly understood and may be important for understanding risk of atrial arrhythmia post-ASD closure. Cardiovascular magnetic resonance (CMR) is an accurate and reproducible imaging modality for the assessment of cardiac function and volumes. We assessed cardiac volumes pre- and post-percutaneous ASD closure using CMR.

**Methods:**

Consecutive patients (n = 23) underwent CMR pre- and 6 months post-ASD closure. Steady state free precession cine CMR was performed using contiguous slices in both short and long axis views through the ASD. Data was collected for assessment of left and right atrial, ventricular end diastolic volumes (EDV) and end systolic volumes (ESV). Data is presented as mean ± SD, volumes as mL, and paired t-testing performed between groups. Statistical significance was taken as p < 0.05.

**Results:**

There was a significant reduction in right ventricular volumes at 6 months post-ASD closure (RVEDV: 208.7 ± 76.7 vs. 140.6 ± 60.4 mL, p < 0.0001) and RVEF was significantly increased (RVEF 35.5 ± 15.5 vs. 42.0 ± 15.2%, p = 0.025). There was a significant increase in the left ventricular volumes (LVEDV 84.8 ± 32.3 vs. 106.3 ± 38.1 mL, p = 0.003 and LVESV 37.4 ± 20.9 vs. 46.8 ± 18.5 mL, p = 0.016). However, there was no significant difference in LVEF and LV mass post-ASD closure. There was a significant reduction in right atrial volumes at 6 months post-ASD closure (pre-closure 110.5 ± 55.7 vs. post-closure 90.7 ± 69.3 mL, p = 0.019). Although there was a trend to a decrease in left atrial volumes post-ASD closure, this was not statistically significant (84.5 ± 34.8 mL to 81.8 ± 44.2 mL, p = NS).

**Conclusion:**

ASD closure leads to normalisation of ventricular volumes and also a reduction in right atrial volume. Further follow-up is required to assess how this predicts outcomes such as risk of atrial arrhythmias after such procedures.

## Background

Atrial septal defects (ASDs) are the most common congenital cardiac malformation first diagnosed in adults and account for approximately 10% of all congenital heart lesions [1,2]. Patients with a significant shunt experience symptoms over time with effort dyspnoea seen in about 30% of patients by the third decade and in over 75% of patients by the fifth decade [3]. The natural course of untreated atrial septal defects often leads to a shortened life expectancy compared to healthy subjects [3]. Longstanding right heart, pulmonary arterial and venous volume overload and dilatation in the setting of an ASD may lead to the development of right heart failure, arrhythmia, thromboembolic events, pulmonary vascular obstructive disease [3-5]. In adults with an ASD and chronic right atrial volume overload, there may be long-term arrhythmias which do not seem to be affected by closure of the defect [6]. In patients aged greater than 40 years with atrial septal defects closed surgically, 60% of these patients develop atrial arrhythmias late after surgery [7]. This may be contributed by chronic right atrial stretch which causes electrophysiological alterations that persist beyond ASD closure [8].

Surgical repair of atrial septal defects has previously been shown to have excellent results in both medium and long term studies [9]. In one retrospective study of patients over 40 years of age with isolated atrial septal defects, surgical repair increased long term survival, with adjusted 10 year survival rate of 95% compared to 84% in the medically treated group [10]. In addition, surgical treatment prevented functional deterioration due to heart failure. However, surgical repair is associated with significant morbidity from peri-operative complications such as pleural effusions, sepsis and pericardial tamponade [11], although the long-term outcome after surgical ASD closure at a young age is much better than surgical closure at adult age [9].

Many adults with secundum ASDs are now able to have these defects closed percutaneously using septal occluder devices such as with the Amplatzer Septal Occluder (ASO), a self-expanding circular double disc that has a conjoint waist containing polytetrafluoroethylene (PTFE) and a nitinol mesh. This device has become an accepted alternative to surgical repair with studies comparing ASO device closure to surgical closure showing decreased complication rates, shorter hospital stays and greater cost-effectiveness [12]. Percutaneous closure of atrial septal defects should reduce right heart volumes by removing left-to-right shunting and thus, lead to symptomatic improvement and increased exercise capacity [12-14]. However, the effects on left ventricular volumes and function are less well described. Ventricular interdependence is a described phenomenon and suggests that a left ventricular effect may well be expected post-ASD closure. Furthermore, atrial changes post-ASD closure have been poorly understood and it is unclear whether there is a significant decrease in atrial arrhythmia post-ASD closure. Thus, atrial changes post-closure may be important for understanding the risk of atrial arrhythmia post-ASD closure.

Cardiovascular magnetic resonance (CMR) is an accurate and reproducible imaging modality for the assessment of cardiac function and volumes. Thus, we sought to use CMR to assess ventricular and atrial volumes directly to document structural changes post-ASD closure.

## Methods

### Subjects

Consecutive patients with secundum atrial septal defects diagnosed on transthoracic and transoesophageal echocardiography with significant left to right shunt (>1.5:1) underwent CMR pre-ASD closure and 6 months post- ASD closure with the Amplatzer Septal Occluder. Only patients in sinus rhythm pre-ASD closure were included.

### CMR

All CMR studies were performed with subjects in the supine position using a 1.5 Tesla MRI scanner (Siemens Sonata, Germany) and a phased array surface coil. For the ventricular image set, long-axis reference views were used for positioning 8 to 12 perpendicular ventricular short-axis slices from the level of the mitral valve to the left ventricular apex. Images were obtained during end-expiratory breath-hold (8 to 10 seconds) with retrospectively ECG-gated True-FISP (Fast Imaging with Steady-State Precession) sequences (Image matrix 256 × 150, field of view 380 mm, repetition time 52.05 ms, echo time 1.74 ms and flip angle 70°). Ventricular short axis slice thickness was 6 mm with intersection gaps of 4 mm, based on previous published studies [15,16]. For the atrial image set, multiple contiguous slices in both short (bi-atrial) and horizontal long axis views (four chamber) were obtained through the ASD, with slice thickness of 6 mm and no intersection gap.

### CMR analysis

Ventricular and atrial analyses were performed off-line with a proprietary software program (Argus software, Siemens Medical Solutions, Germany). For the left ventricular (LV) data set, short-axis endocardial and epicardial contours were manually traced in end-diastole (at start of R-wave) and in end-systole (smallest cavity area). Papillary muscles and trabeculations were excluded from the ventricular volume and were included if contiguous with the myocardial mass. The basal slice was selected as the slice where the blood volume was surrounded by >50% of ventricular myocardium [15,16]. For the right ventricular (RV) data set, one observer manually traced the endocardial contours at end-diastole and at end-systole. The selection of the RV basal slice was based on published methods where, if the pulmonary valve was seen, only the portion of the volume below the level of the pulmonary valve was included [16]. Both LV and RV end-diastolic and end-systolic cavity surface areas were summed up and volumes: end-diastolic (EDV) and end-systolic (ESV) estimated by multiplying with interslice intervals as per Simpson's rule. Ejection fraction (EF) was calculated as EF = (EDV-ESV)/EDV × 100 (%) and left ventricular mass as LV mass = 1.05 × (epicardial volume - endocardial volume).

A true three-dimensional atrial volume assessment technique was used, in which the endocardial borders were manually traced for both left and right atria in the horizontal long axis (four chamber) views in ventricular end-systole when atrial volume is maximal [17]. Borders of left atrium were defined as the plane of the mitral valve and the visually apparent juncture of left atrium with pulmonary veins. Borders of right atrium were defined as the plane of the tricuspid valve and the juncture with the vena cavae. The atrial appendage was included if present on the images. Atrial volumes were calculated as a sum of atrial cavity areas with interslice intervals using a modification of Simpson's rule.

### ASD device implantation

All ASDs were closed with the Amplatzer Septal occluder (AGA medical corporation). Procedures were performed under general anaesthesia with transoesophageal echocardiographic guidance and fluoroscopy. A sizing balloon was used to determine the stretched diameter of the ASD before selection and deployment of ASO device, as previously described [18]. In brief, a Meditech balloon (Boston Scientific, Watertown, MA) sized 20 or 27 mm in this series is used. This balloon is inflated within the left atrium and firm continuous pressure applied to pull it into the atrial septum, using TOE guidance. The diameter at which the balloon just gets through the atrial septal defect is the stretched balloon diameter (SBD).

### Follow-up CMR

**Figure 1:**
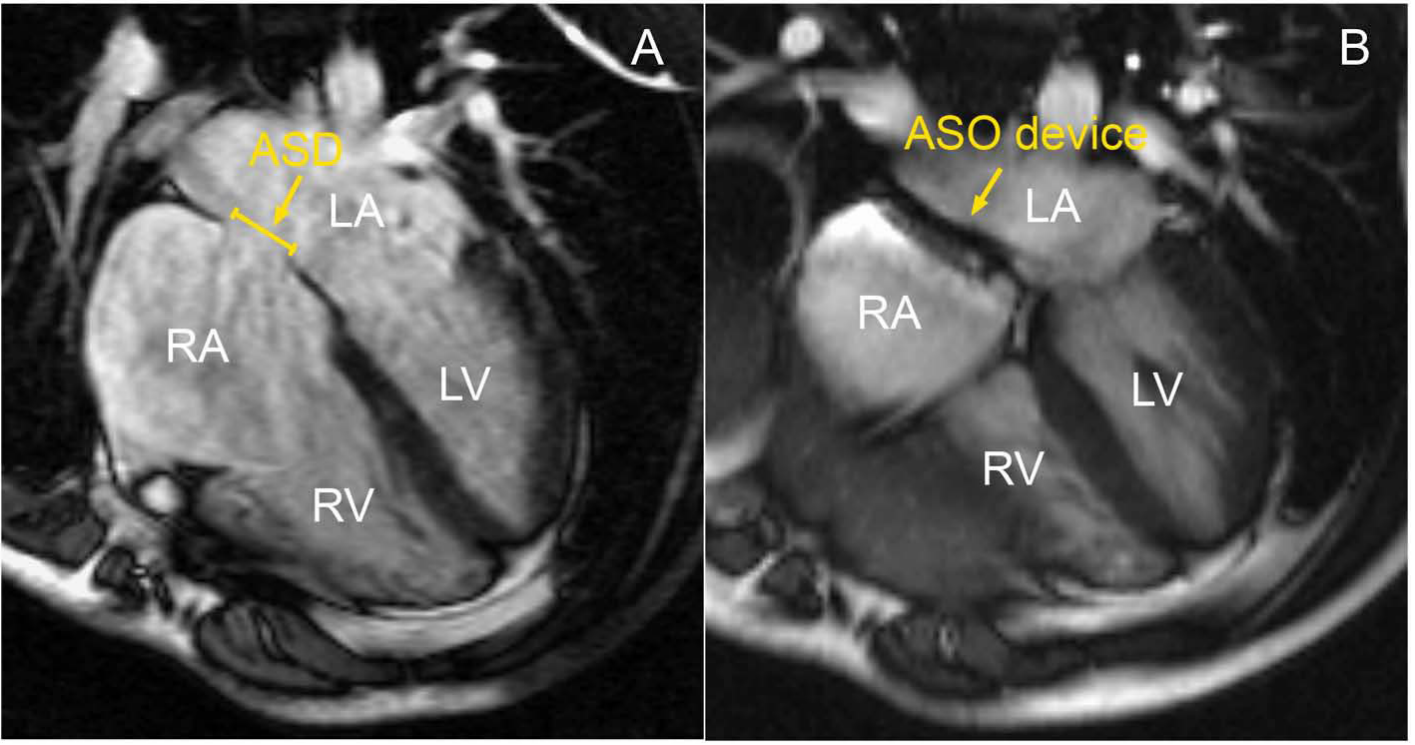
Four chamber CMR views showing the ASD pre- (panel A) and post-closure with the Amplatzer Septal Occluder (ASO) device (panel B). RA = right atrium, LA = left atrium, RV = right ventricle and LV = left ventricle.

**Figure 2:**
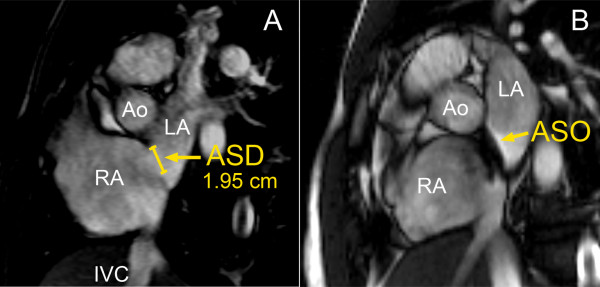
Bi-atrial short axis views showing the ASD (same patient as Figure 1) pre- (panel A) and post-closure with the ASO (panel B). RA = right atrium, LA = left atrium, Ao = aorta and IVC = inferior vena cava).

CMR was repeated at 6 months from the time of the ASD closure, with the full dataset obtained to re-assess cardiac volumes and function (see Figures 1 and 2).

### Reproducibility

Intra- and inter- observer variability was assessed for the left and right atrial and ventricular parameters.

### Statistical analysis

Data is presented as mean ± standard deviation. Comparison of cardiac volumes pre- and 6 months post-ASD closure was performed with paired t testing between groups. Statistical significance was taken as p < 0.05. Inter and intraobserver variability was assessed using the Bland and Altman method [19] as well as intraclass correlation coefficients.

## Results

A total of 23 patients were included in the study (mean age 51.2 ± years, 20 females and 3 males). Two patients who went on to surgical closure of their ASDs due to size of the defect were excluded from the analysis and one patient did not attend the follow up scan. Follow up CMR studies were obtained in 20 patients.

### Atrial changes

There was a significant reduction in right atrial volumes at 6 months post-ASD closure (pre-closure 110.5 ± 55.7 vs. post-closure 90.7 ± 69.3 mL, p = 0.019). Although there was a trend to a decrease in left atrial volumes post-ASD closure, this was not statistically significant (84.5 ± 34.8 mL to 81.8 ± 44.2 mL, p = NS).

### Ventricular changes

There was a significant reduction in right ventricular volumes at 6 months post-ASD closure (RVEDV: 208.7 ± 76.7 vs. 140.6 ± 60.4 mL, p < 0.0001) and RVEF was significantly increased (RVEF 35.5 ± 15.5 vs. 42.0 ± 15.2%, p = 0.025). Left ventricular volumes were significantly increased (LVEDV 84.8 ± 32.3 vs. 106.3 ± 38.1 mL, p = 0.003 and LVESV 37.4 ± 20.9 vs. 46.8 ± 18.5 mL, p = 0.016). There was no significant difference in LVEF and LV mass post-ASD closure (See Table 1).

**Table 1:** CMR data pre and post-percutaneous ASD closure.

	**Pre-closure**	**Post-Closure**	**Change (%)**	**P-value**
**RV EDV (mL)**	208.7 ± 76.7	140.6 ± 60.4	↓ 32.6	< 0.0001
**RV ESV (mL)**	134.0 ± 63.7	81.6 ± 41.5	↓ 39.1	< 0.0001
**RV EF (%)**	35.5 ± 15.5	42.0 ± 15.2	↑ 18.3	0.025
**LV EDV (mL)**	84.9 ± 32.3	106.3 ± 38.1	↑ 25.2	0.003
**LV ESV (mL)**	37.3 ± 20.9	46.8 ± 18.5	↑ 25.4	0.016
**LV EF (%)**	61.7 ± 12.8	58.2 ± 9.0	↓ 5.7	NS
**LV mass (g)**	91.2 ± 29.5	103.0 ± 48.0	↑ 12.9	NS
**RA vol. (mL)**	110.5 ± 55.7	90.7 ± 69.3	↓ 17.9	0.019
**LA vol. (mL)**	84.5 ± 34.8	81.8 ± 44.2	↓ 3.2	NS

RV = right ventricle, LV = left ventricle, RA = right atrial, LA = left atrial, EDV = end-diastolic volume, ESV = end-systolic volume, EF = ejection fraction, vol. = volume, NS = non-significance.

Indexed right ventricular volumes (to body surface area) pre-ASD closure were greater than aged-matched controls at baseline (RV EDV/BSA 130.1 ± 23.8 vs. 65.9 ± 15.9, p < 0.0001 and RV ESV/BSA 77.7 ± 31.1 vs. 33.9 ± 24.1, p < 0.0001), but were not significantly different to aged-matched controls post-ASD closure (RV EDV/BSA 84.6 ± 36.3 vs. 65.9 ± 15.9, p = 0.06 and RV ESV/BSA 47.6 ± 24.1 vs. 33.9 ± 16.5, p = 0.07). Indexed left ventricular volumes and mass were not significantly different to aged-matched controls pre- or post- ASD closure (See Table 2).

**Table 2:** Comparison of indexed RV and LV volumes to aged-matched controls

	**Pre-closure (mL/m^2^)**	**Post-closure (mL/m^2^)**	**Aged-match (mL/m^2^)**	**Unpaired t-test (vs. post-closure)**
**RV EDV/BSA**	130 ± 32.8	84.6 ± 36.3	65.9 ± 15.9	0.07
**RV ESV/BSA**	77.7 ± 31.1	47.6 ± 24.1	33.9 ± 16.5	0.07
**LV EDV/BSA**	51.8 ± 12.6	64.8 ± 16.1	59.6 ± 14.3	NS
**LV ESV/BSA**	21.0 ± 10.8	26.4 ± 10.2	25.9 ± 8.1	NS
**LV mass/BSA**	50.6 ± 13.4	58.6 ± 29.5	49.8 ± 9.1	NS

### Reproducibility

#### Interobserver variability

Intraclass correlation coefficients between 2 independent observers for RV and LV parameters were: RV EDV 0.93, RV ESV 0.89, LV EDV 0.96, LV ESV 0.89, LV mass 0.84, RA volume 0.92, LA volume 0.87, RV EF 0.70, LVEF 0.94. Results from the Bland and Altman analyses for interobserver variability for LV and RV are presented as mean, the difference between the means ± 2 SD: LV EDV mean 86.2, difference 6.1 ± 22.6 mL, LV EF mean 62.9, difference -4.8 ± 24.6%, RVEDV mean 211.0, difference 19.6 ± 57.4 mL and RV EF mean 43.2, difference 10.1 ± 15.6%. Interobserver mean and difference between means of atrial volumes ± 2 SD were: LA volume 146.8, difference -2.2 ± 25.5 mL and RA volume mean 128.7, difference -4.7 ± 40.4 mL.

#### Intraobserver variability

Intraclass correlation coefficients for intraobserver variability for RV and LV parameters were RV EDV 0.89, RV ESV 0.85, LV EDV 0.95, LV ESV 0.98, RA volume 0.98, LA volume 0.84, RV EF 0.77, LV EF 0.91.

## Discussion

We have shown that percutaneous ASD closure leads to a reduction in RV volumes, in association with an increase in LV volumes by CMR. This was associated with an improvement in RVEF, but no change in LVEF. Furthermore, for the first time, we have seen a significant decrease in right atrial volumes post-ASD closure by CMR, but a non-significant change in left atrial volumes.

In our study, we have used CMR to obtain a full volume data set for both ventricles and atria. CMR has been shown to be safe post-cardiovascular implants [20] and has become the gold standard in the assessment of left and right ventricular volumes, function and mass with excellent accuracy and reproducibility [15,16,21,22]. Furthermore, compared to echocardiography, CMR has the advantage in the assessment of right ventricular volumes, given the non-geometric shape of the right ventricle, which makes quantification of right ventricular volume difficult by echocardiography. Previous studies on the effect of ASD closure on chamber volumes have mainly been performed using echocardiography although more recently, there have been a couple of studies that have looked at atrial and ventricular changes using CMR [23,24].

Atrial assessments of atrial dimensions using a one or two-dimensional parameter or estimation of atrial volume based on two-dimensional image algorithms have limitations. Echocardiography M-mode atrial dimension as a measure of atrial size assumes that a direct relationship between this one-dimensional measurement and atrial volume. Biplane two-dimensional echocardiography assumes an elliptical geometry of the atria [25]. These clearly are not exact, due to the variable and non-uniform geometry of the atria with significant differences demonstrated in the measurement of atrial volumes using biplane area length and biplane modified Simpson methods [26]. Atrial enlargement may also be asymmetrical. CMR volume assessment of the atrium with cine imaging requires no assumptions about the geometry of the atria and has become an accepted standard for the determination of atrial volumes [27-29]. We therefore obtained a true three-dimensional atrial volume assessment technique in which the atrial borders were traced for both left and right atria in the horizontal long axis (four chamber) views in ventricular end-systole when atrial volume is maximal [17].

### ASD closure

Echocardiography studies in adult and paediatric patients with closure of atrial septal defects have shown a reduction in right atrial areas and ventricular volumes after ASD closure both surgically [30] and percutaneously [24,31-35]. However, there is a subgroup of patients where right atrial and right ventricular sizes do not normalize. This has been reported in adult patients of 28% for RA [34] and 29% for RV 1 year after percutaneous closure [33]. Persistent right atrial and ventricular enlargement is associated with higher age at closure [31-34]. However, even in the group of patients who had ASD closure during childhood, there was a group (>20%) with persistent RV dilatation and RA enlargement [9].

RV ejection fraction has been shown to decrease after surgical ASD repair. The possible causes of this were thought to include a true reduction in RV ejection fraction post-operatively, less volume overload of the right ventricle, less paradoxical septal motion or an inaccuracy in echocardiography methods for evaluating RV ejection fraction [30]. RV systolic and diastolic function may be supra normal due to the increased pulmonary blood flow, while those of the left ventricle may be within normal range [36]. No changes were found in left ventricular (LV) volumes and ejection fraction in this study [30]. Global LV function has been shown to improve after percutaneous ASD closure. In a recent study, myocardial performance index (MPI), a measure of combined systolic and diastolic function [37] was significantly improved for both RV and LV [38] in patients who underwent percutaneous ASD closure. The same index measured post-surgical ASD closure has been shown to be not significantly different [37] and previous investigators have indicated that this may be contributed to by cardiopulmonary bypass [30,36], which is avoided by percutaneous closure.

### Percutaneous ASD closure

Cardiac remodelling post-ASD closure has been shown to be an early event, with reduction in right ventricular and atrial sizes occurring in the first week post-closure and that after 4 months no changes were observed [39]. Significant changes also occurred in the left ventricle, with increase in LV dimensions post-closure, but without changes in LA dimensions.

Because of its shape, quantitative echocardiographic methods to evaluate RV volumes have been difficult and thus limited to 2-D measurements. Right-sided cardiac parameters measured have included RV and RA diameters, RVOT diameters and the assessment of paradoxical septal motion [13,32,33,38]. Even at one month post-ASD closure, mean RV size and mean RVOT diameter was significantly reduced to within normal limits while mean right atrial length showed a trend toward shortening at one month and reached significance at 6 months [33]. At one year, a third of patients demonstrated persistent RV enlargement [33]. Our data with more accurate assessment of right atrial dimensions clearly shows a volume reduction in all patients.

Closure of secundum ASD results in decreased indexed RV volume (as measured by transthoracic echocardiography) comparable to that in control subjects at 24 months following closure. However indexed RA area remained greater than in the control group. Decrease in indexed RA area over the first 12 months of follow-up was related to young age at time of closure [31].

Global LV function has been shown to improve after percutaneous ASD closure. In a recent study, myocardial performance index (MPI), a measure of combined systolic and diastolic function [37] was significantly improved for both RV and LV [38] in patients who underwent percutaneous ASD closure. The same index measured post-surgical ASD closure has been shown to be not significantly different [40] and previous investigators have indicated that this may be contributed to by cardiopulmonary bypass [30,36], which is avoided by percutaneous closure. In our data set however, there was no significant change in LVEF although both LVEDV and LVESV increased.

### Interventricular dependence

Ventricular interdependence has been well described in echocardiography studies and highlights the important role of the inter-ventricular septum in RV and LV function. In RV volume overload, the septum bulges into and encroaches on the LV cavity and leads to impairment of LV filling. The right ventricle is also more compliant than the left ventricle so the left ventricle is relatively under filled. The mechanisms for decreased LV performance associated with RV volume overload include the mechanical disadvantage of a non-circular short axis configuration and changes in chamber and myocardial preload [41].

Reduced LV systolic function associated with RV volume overload has been described [42-44]. One echocardiographic study has reported the occurrence of adverse ventricular interdependence associated with RV volume overload in patients with ASDs [41] in which the septum bulges into and encroaches on the LV cavity and leads to impairment of LV filling. Since the right ventricle is also more compliant than the left ventricle, the left ventricle is relatively under filled. However, the reversibility of this ventricular dysfunction after elimination of the RV volume overload with surgical closure has not been consistently demonstrated [43]. This may reflect surgical confounding factors that affect LV function, including pericardectomy, cardiopulmonary bypass and postoperative alterations in preload, afterload and heart rate [41].

Percutaneous device closure of the ASD avoids the confounding factors caused by surgery. In a study of 34 patients, LV ejection fraction obtained by echocardiography improved significantly from 54.9 to 62.1% (P < 0.001) [41].

In our study, left ventricular volumes were reduced as compared to normal values, but increased 6 months post-closure of the ASD. This is similar to a recent study which looked at left ventricular dimensions with CMR post-ASD closure [45]. In addition, they found NT-proBNP concentrations were within the normal range pre-ASD closure, but increased early after interventional closure and was associated with the increase in left ventricular dimensions as assessed by CMR [45]. However, they failed to evaluate atrial volumetric changes pre- and post- ASD closure.

### Limitations

In this study, formal assessment of shunt calculation by Qp:Qs was not performed on the baseline CMR studies initially. Therefore, we were unable to assess any relationships between degree of left to right shunting with changes in cardiac volumes after percutaneous ASD closure.

## Conclusion

Percutaneous ASD closure leads to normalisation of both RV and LV volumes using CMR. With a true volumetric CMR technique, we note that RA volume reduces significantly although the small LA volume reduction was not significant. This may have important implications for future risk of atrial arrhythmogenicity.

## Competing interests

The authors declare that they have no competing interests.

## Authors' contributions

KST participated in the study design, CMR data acquisition, analysis and manuscript preparation. BKD and AC participated in data analysis and manuscript revision. PM and KW were involved in CMR acquisition. MB, MIW, PJD, PS contributed to manuscript revision. SGW conceived the study, supervised the CMR acquisition and revised the manuscript. All authors read and approved the final manuscript.
